# bFGF regulates autophagy and ubiquitinated protein accumulation induced by myocardial ischemia/reperfusion via the activation of the PI3K/Akt/mTOR pathway

**DOI:** 10.1038/srep09287

**Published:** 2015-03-19

**Authors:** Zhou-Guang Wang, Yue Wang, Yan Huang, Qin Lu, Lei Zheng, Dong Hu, Wen-Ke Feng, Yan-Long Liu, Kang-Ting Ji, Hong-Yu Zhang, Xiao-Bing Fu, Xiao-Kun Li, Mao-Ping Chu, Jian Xiao

**Affiliations:** 1 School of Pharmacy, Key Laboratory of Biotechnology and Pharmaceutical Engineering, Wenzhou Medical University, Wenzhou 325035, China; 2 Department of Biochemistry and Molecular Biology, College of Basic Medical Science, Jilin University, Changchun, 130012, China; 3 Department of Pediatric Cardiology, The Second Affiliated Hospital, Wenzhou Medical University, Wenzhou, 325000, China; 4 Department of Ultrasound, The Second Affiliated Hospital, Wenzhou Medical University, Wenzhou, 325000, China; 5 Department of Medical Immunology, Medical School, Anhui University of Science and Technology, Huainan 232001, China; 6 Institute of Basic Medical Science, Chinese PLA General Hospital, Beijing 100853, China

## Abstract

Autophagy is involved in the development and/or progression of many diseases, including myocardial ischemia/reperfusion (I/R). In this study, we hypothesized a protective role of basic fibroblast growth factor (bFGF) both *in vivo* and *in vitro* and demonstrated that excessive autophagy and ubiquitinated protein accumulation is involved in the myocardial I/R model. Our results showed that bFGF improved heart function recovery and increased the survival of cardiomyocytes in myocardial I/R model. The protective effect of bFGF is related to the inhibition of LC3II levels. Additionally, bFGF enhances the clearance of Ub by p62 and increases the survival of H9C2 cells. Moreover, silencing of p62 partially blocks the clearance of Ub and abolishes the anti-apoptosis effect of bFGF. An shRNA against the autophagic machinery Atg7 increased the survival of H9C2 cells co-treated with bFGF and rapamycin. bFGF activates the downstream signaling of the PI3K/Akt/mTOR pathway. These results indicate that the role of bFGF in myocardial I/R recovery is related to the inhibition of excessive autophagy and increased ubiquitinated protein clearance via the activation of PI3K/Akt/mTOR signaling. Overall, our study suggests a new direction for bFGF drug development for heart disease and identifies protein signaling pathways involved in bFGF action.

Myocardial ischemia/reperfusion (I/R) has high morbidity and mortality and is a significant burden for patients and society. Despite recent progress in clinical interventions to facilitate early myocardial reperfusion for patients who suffer from acute myocardial infarction, the death rate during the acute phase of myocardial infarction is approximately 10% and the incidence of heart failure reaches 25% during the chronic phase^1^. The current standard treatment for myocardial ischemia is rapid reperfusion, which can attenuate myocardial infarction, reduce cardiomyocyte apoptosis and restore contractile dysfunction. However, reperfusion potentially causes additional injury, disturbances in ionic homeostasis, local edema, ischemia, focal hemorrhage, free radical stress and inflammatory responses. Many studies reported that autophagy also plays a key role in myocardial I/R injury in both animal models and cellular models by causing progressive degeneration of the heart^2^,^3^.

Autophagy is a dynamic process that turns over organelles and proteins through a lysosome-associated degradation process, and serves a critical function in cellular homoeostasis by regulating cell survival and cell death pathways^4^. In addition to its role in cellular homeostasis, autophagy may play a cytoprotective role in instances of nutrient starvation^5^ or a specific type of programmed cell death^6^. The functional role of autophagy in myocardial I/R is currently under intense investigation, and prior studies have characterized this process both *in vitro* and *in vivo*. Interestingly, up-regulation of autophagy has been reported to both contribute to and cause cell death in the heart^7^,^8^. The ubiquitin-proteasome system (UPS) plays specific roles in the heart and leads to the accumulation of misfolded proteins that may further exacerbate cardiac diseases^9^. The ubiquitin-binding protein, p62, also known as sequestome-1/SQSTM1^2^,^8^, a multifunctional, multi-domain scaffolding protein that serves as a hub for several signaling pathways, including autophagy-mediated protein degradation, nuclear factor kappa-B (NF-κB) activation, and nuclear factor erythroid 2-related factor (NRF2) activation^8^,^9^. p62/SQSTM1 (sequestosome 1) is a multifunctional adaptor protein^10^,^11^, and the C-terminal of p62 is a ubiquitin-associated domain, which interacts with ubiquitinated proteins and targets them to the microtubule-associated protein 1 light chain 3-II (LC3-II), where they are then selectively degraded by autophagy^12^,^13^. p62 promotes survival-critical signals, including proliferation, differentiation and induction of anti-apoptotic genes^14^,^15^. p62 is considered a linking protein between ubiquitinated proteins and autophagosomes, and it facilitates the maintenance of cell homeostasis and survival^16^. The underlying mechanisms of recovery in myocardial I/R and the role of p62 in this process are remain unclear.

Basic fibroblast growth factor (bFGF or FGF-2) is a member of the fibroblast growth factors (FGFs) that regulate a variety of biological functions including proliferation, morphogenesis and suppression of apoptosis during development via a complex signal transduction system^17^,^18^. bFGF is highly expressed in the nervous system and heart, where it has multiple roles, and it has previously been shown to support the survival and growth of cultured cardiomyocytes. Several growth factors have cardioprotective effects and improve recovery in myocardial I/R. In particular, bFGF transient infusion or transgene therapy may promote cardiovascular and functional recovery^19^. Moreover, recent studies have shown a definite relationship between FGF signals and autophagy through the interaction of the mammalian target rapamycin (mTOR), which is positively regulated by the PI3K/Akt signaling pathway^20^,^21^. However, the molecular mechanism of bFGF treatment in recovery during myocardial I/R has not been completely defined. Specifically, the relationship between autophagy and ubiquitination and the therapeutic effect of bFGF in the heart has not been previously investigated.

In this study, we examined bFGF-mediated effects in cardioprotection, functional recovery, autophagic activity, the accumulation of ubiquitinated proteins and the involvement of downstream signals following myocardial I/R using histologic and protein analyses. To the best of our knowledge, these findings are the first to identify a link between bFGF and autophagy-mediated ubiquitinated protein degradation and the PI3K/Akt/mTOR signaling after myocardial I/R. Collectively, our results suggest that the bFGF may be an effective and feasible target for drug development for myocardial diseases both *in vivo* and *in vitro.*

## Results

### bFGF improves cardiac function after myocardial I/R

To evaluate the therapeutic effect of bFGF in cardiac function after myocardial I/R, 2-D echocardiographic was used to monitor cardiac contractility. After 3 days, the LVEDd and LVESd of the control group increased to 3.4 ± 0.2 mm^2^ and 2.1 ± 0.3 mm^2^ (Table 1), which are statistically higher than the normal animals, and indicated that post-MI led to ventricular dilation. By contrast, bFGF treated animals had lower LVEDd value of 3.1 ± 0.1 mm^2^ and LVESd values of 1.6 ± 0.1 mm^2^ compared to the myocardial I/R groups. The left EF decreased significantly from 92.0 ± 2.3 in the control group to 72.9 ± 1.4 in the animal model group. After bFGF treatment, the EF reversed to 83.1 ± 2.5. Compared to the left FS, both have a similar result for bFGF treatment. In summary, the result of the echocardiography demonstrated that bFGF effectively improved cardiac function after myocardial I/R.

### bFGF decreases myocardial apoptosis and fibrosis in myocardial I/R

**Figure 1:**
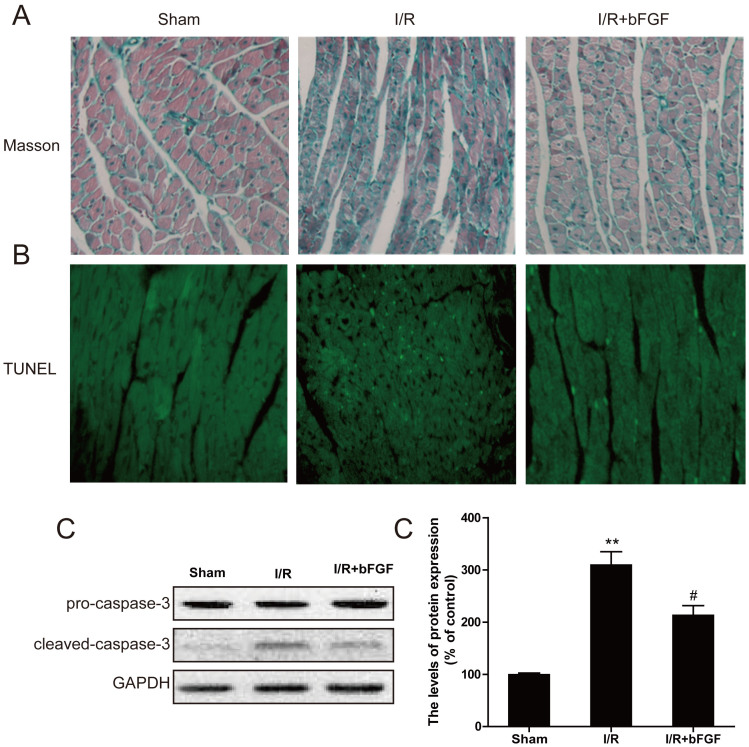
bFGF reduces myocardial apoptosis and fibrosis in the hearts of mice after myocardial I/R. (A) Fibrosis in the border zone at 3 days as examined by Masson's trichrome staining. (B) Representative immunofluorescent TUNEL of sections from the ischemic area in the hearts of mice that received bFGF or vehicle. (C) Caspase-3 was detected by western blotting. The protein expression of cleaved-caspase-3 in the hearts of sham mice, I/R mice and I/R mice treated with bFGF. (D) The optical density analysis of cleaved-caspase-3 in the heart. The mean values ± SEM, n = 6 per group. * *P* < 0.05, ** *P* < 0.01, versus the sham group, ^#^ represents *P* < 0.05, ^##^ *P* < 0.01 versus the I/R group. I/R, ischemia/reperfusion.

To assess myocardial apoptosis, TUNEL staining was performed in each group. As shown in Figure 1A, there are no TUNEL-positive cells in the control group. The numbers of TUNEL-positive cells increased significantly after I/R for 1 d, and bFGF showed significant protective effects with fewer myocardial apoptotic cells (Fig. 1A). The protein expression of caspase-3 in the heart after myocardial I/R was detected by western blot analysis. The expression of cleaved-caspase-3 protein significantly decreased after bFGF treatment compared to myocardial I/R model after 1 d injury (Fig. 1C and 1D).

Reports indicated that myocardial ischemia presents with obvious myocardial necrosis and fibrosis, and our masson staining result also showed that I/R caused collagen deposition in the infarct area. In the control group, fibrosis extended to the border zone as indicated by dense collagen deposition along the ventricular wall. By contrast, bFGF reduced the collagen content in the border zone (Fig. 1B). These data indicate that bFGF protects the myocardium by reducing apoptosis and fibrosis.

### The Protective Effect of bFGF is related to inhibited autophagy in the myocardial I/R

**Figure 2:**
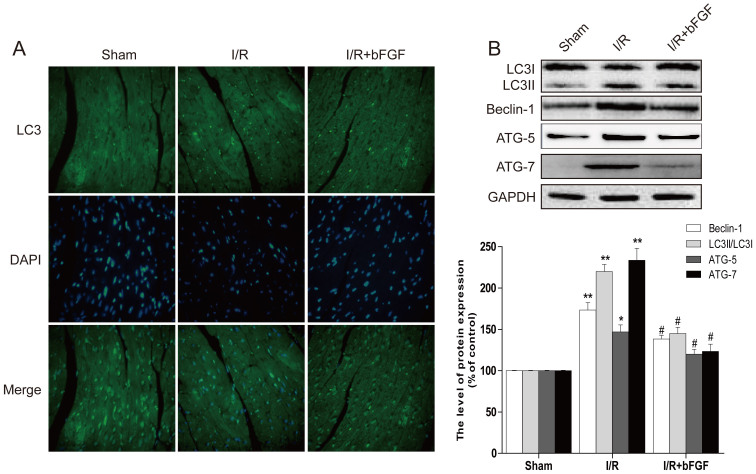
bFGF decreases the level of LC3II/LC3I in heart lesions at 3 days after I/R. (A) Immunofluorescence staining results of LC3 (green), the nuclei were labeled with Hoechst (blue). (B) The protein expression of LC3, beclin-1, ATG-7 and ATG-5 in the sham, I/R model group and I/R model mice treated with bFGF. ** *P* < 0.01 versus the sham group, ^#^ *P* < 0.05 versus the I/R group. The mean values ± SEM, n = 6.

Autophagy activation is involved in myocardial I/R; however, the role of bFGF in regulating autophagy in myocardial I/R has not been clearly defined. In our model, we first detected the regulation of bFGF to the LC3 protein and beclin-1, which are often used as indicators of autophagy. The immunofluorescence staining results showed that the LC3 positive green dots increased in the heart lesions compared to the sham group. The LC3 positive green dots decreased in the bFGF-treated group, consistent with the western blot results (Fig. 2A). To further investigate the effect of bFGF on autophagy, LC3 and beclin-1 expression in myocardial I/R tissues were detected by western blot analysis. As shown in Figure 2B, LC3II, beclin-1 and the ratio of LC3II/LC3I increased in the myocardial I/R model mice compared to the sham group (*P* < 0.01). This effect was markedly inhibited by bFGF treatment at 4 h after I/R.

### bFGF improves recovery after myocardial I/R by autophagic clearance of Ubiquitinated protein accumulation

**Figure 3:**
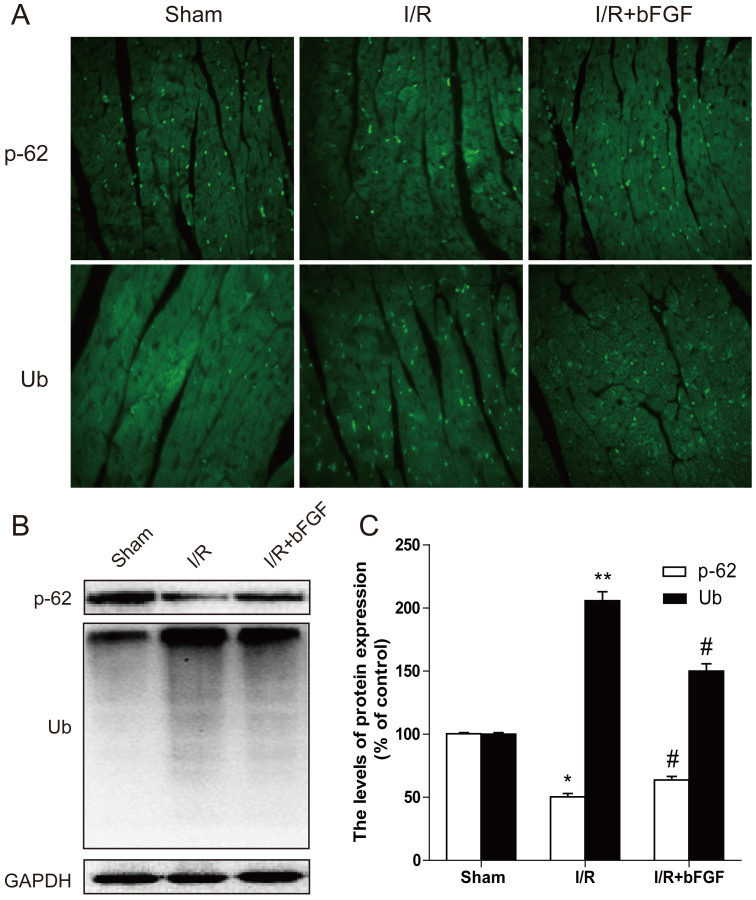
bFGF activates p62 and clears ubiquitinated proteins 3 days after I/R. (A) Immunofluorescence staining results of Ub (green) and SQSTM1/p-62 (green). (B) The protein expression of Ub and SQSTM1/p-62 (Thr269/Ser272) in the sham, I/R model group and I/R model mice treated with bFGF. (C) The optical density analysis of Ub and p62 protein. ** *P* < 0.01 versus the sham group, ^#^ represents *P* < 0.05 versus the SCI group. The mean values ± SEM, n = 6.

As recognition signals for the proteasome, polyubiquitin chains are the major regulator of protein abundance in cells, often initiating proteolysis of substrates. Several lines of evidence indicated that p62 is an ubiquitin-binding protein that acts as a shuttling factor that targets poly-ubiquitinated proteins for degradation by either autophagy or proteasome pathways^8^,^9^. To determine whether bFGF also impacts protein degradation and homeostasis, the protein expression levels of poly-ubiquitinated proteins and p62 were determined using western blotting. As demonstrated in Figure 3B, poly-ubiquitinated protein expression increased and p62 expression decreased after myocardial I/R injury. After bFGF treatment at 4 h post-reperfusion, the levels of poly-ubiquitinated protein decreased significantly, whereas p62 expression was increased (*P* < 0.05). The immunofluorescence staining results demonstrated that poly-ubiquitinated protein expression increased significantly in rat heart lesions, whereas p62 expression was decreased. Additionally, bFGF down-regulated the accumulation of poly-ubiquitinated protein and increased the expression of p62 (Fig. 3A), suggesting that the effect of bFGF in recovery after reperfusion injury may also be related to the regulation of autophagic protein degradation mediated by p62.

### Activation of the PI3K/Akt/mTOR signaling pathways is involved in the role of bFGF in myocardial I/R

**Figure 4:**
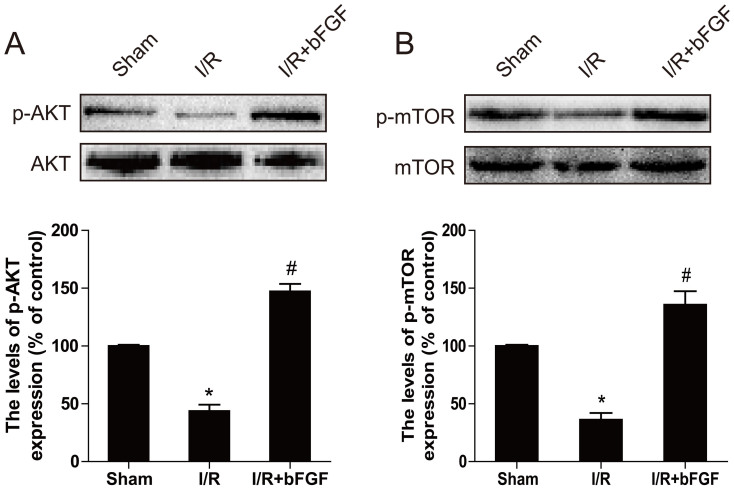
bFGF activates the Akt/mTOR signaling pathways 3 days after ischemia/reperfusion. (A) The protein expression of p-Akt (Ser473)/Akt, p-mTOR (Ser2448)/mTOR in the sham, I/R model group and I/R model mice treated with bFGF. (B) The optical density analysis of the p-Akt/Akt and p-mTOR/mTOR protein. ** *P* < 0.01 versus the sham group, ^#^ *P* < 0.05 versus the SCI group. The mean values ± SEM, n = 6.

The PI3K/Akt signaling pathways are essential for the role of bFGF in cardiomyocyte proliferation and differentiation. mTOR is an upstream signal in the regulation of autophagy, which is positively regulated by PI3K/Akt and results in the inhibition of autophagy. Recent evidence suggested an effect of Akt/mTOR signaling in the regulation of autophagy in cardiac stem cells; however, whether this signal pathway is mediated by bFGF in myocardial I/R has not been assessed. We first detected the regulation of bFGF to the Akt/mTOR signaling pathways in our I/R model *in vivo*. Western blot analysis demonstrated that the phosphorylation of Akt/mTOR significantly decreased after myocardial I/R injury in mice (Fig. 4A and 4B). Reduced phosphorylation levels were obtained after bFGF treatment compared to the I/R group (*P* < 0.05). These data suggest that recovery from myocardial I/R with bFGF treatment may occur in part through the activation of Akt/mTOR signaling and inhibition of autophagy.

### Autophagy activator rapamycin partially abolishes the protective effect of bFGF in myocardial I/R

**Figure 5:**
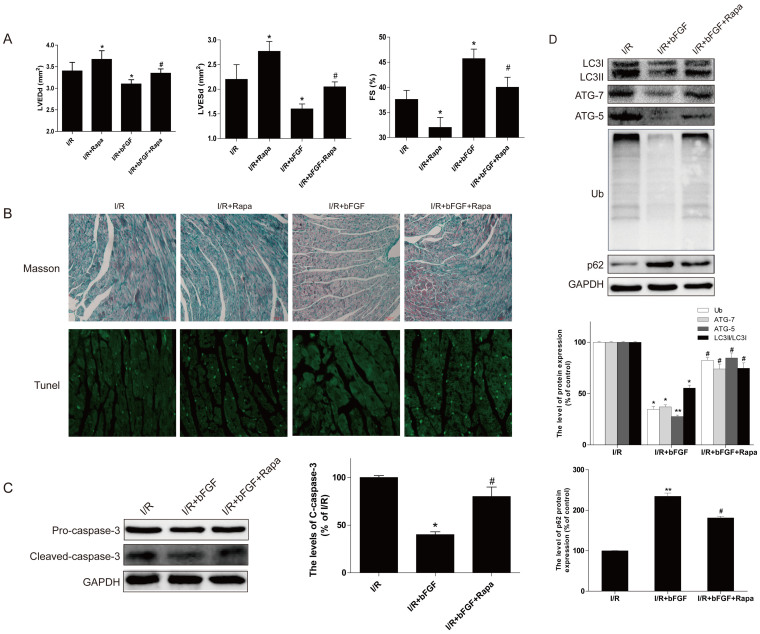
The protective effect of bFGF in I/R is inhibited by rapamycin. (A) The echocardiography analysis of I/R, I/R rats treated with rapamycin, bFGF and bFGF with rapamycin. The left ventricular dimension at end-diastole and end-systole (LVEDd, LVEDs), FS = 100 × (LVEDd – LVEDs)/LVEDd. *P < 0.05 versus the I/R group. (B) Fibrosis in the border zone at 3 days as examined by Masson's trichrome staining of I/R, I/R mice treated with rapamycin, bFGF and bFGF with rapamycin. TUNEL staining of I/R, I/R mice treated with rapamycin, bFGF and bFGF with rapamycin. (C) The optical density analysis of cleaved-caspase-3 in the heart. The mean values ± SEM, n = 6 per group. * *P* < 0.05 versus the I/R group, ^#^ represents *P* < 0.05 versus the I/R+bFGF group. (D) Protein expression of LC3, ATG-7, ATG-5, Ub, and SQSTM1/p-62 (Thr269/Ser272) in I/R mice treated with bFGF and bFGF with rapamycin. The optical density analysis of LC3, ATG-7, ATG-5, Ub, and SQSTM1/p-62 of I/R mice treated with bFGF and bFGF with rapamycin. *P < 0.05, **P < 0.01 versus the I/R group, ^#^ represents *P* < 0.05 versus the I/R+bFGF group. I/R, ischemia/reperfusion.

To further confirm our hypothesis that inhibition of autophagy is important for the protective effect of bFGF in I/R recovery, a classical autophagy sensitizer, rapamycin, was added to the bFGF solution around the I/R areas, which is generally used to induce autophagy. The LVEDd, LVESd and FS results indicated that compared to the mice treated with bFGF alone, bFGF-related recovery in the I/R mice was impaired when treatment was combined with rapamycin (Fig. 5A). The Masson staining results showed that the collagen deposition in the infarct area increased after treatment with rapamycin compared to bFGF alone (Fig. 5B). The TUNEL staining results also showed that there was an extensive loss of cells in the I/R group treated with bFGF and rapamycin compared to the bFGF-treated I/R group (Fig. 5C). Moreover, the western blot results also showed that the expression of cleaved-caspase-3 increased significantly after treated with rapamycin compared to the bFGF treatment group. These results indicate that the addition of rapamycin partially abolished the effect of exogenous bFGF, which further supports the role of bFGF in the recovery of heart I/R.

The expression of LC3II/LC3I, ATG-7, ATG-5, poly-ubiquitinated protein and p62 was evaluated to determine the mechanism of autophagy induction in the cardioprotective effect of bFGF treatment in the heart I/R model. As shown in Fig. 5D and 5E, the levels of LC3II/LC3I, ATG-7, ATG-5, and poly-ubiquitinated protein increased when rapamycin was added to the treatment solution, and p62 protein expression was reduced compared to the bFGF group. These findings suggest that rapamycin induced activation of autophagy and played a negative role in the recovery from heart I/R with bFGF treatment. The inhibition of autophagy and clearance of poly-ubiquitinated proteins contributed to the cardioprotective effect of bFGF *in vivo*.

### Exogenous bFGF protects H9C2 Cells by inhibiting excessive autophagy *in vitro*

**Figure 6:**
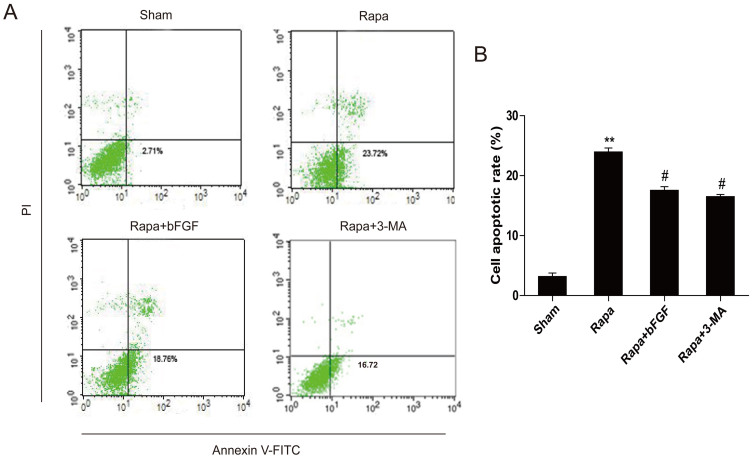
The anti-apoptosis effects of bFGF are related to the inhibition of autophagy, which is induced by rapamycin. (A) H9C2 cells were treated with 100 ng/ml rapamycin with or without 40 ng/ml bFGF. The cells were collected and stained with annexin V-FITC/propidium iodide and detected by flow cytometry. The lower right panel indicates the apoptotic cells. (B) A bar diagram of the apoptotic cell rate from three separate experiments. **P < 0.01 vs. the control; ^#^*P* < 0.05 vs. the rapamycin group.

**Figure 7:**
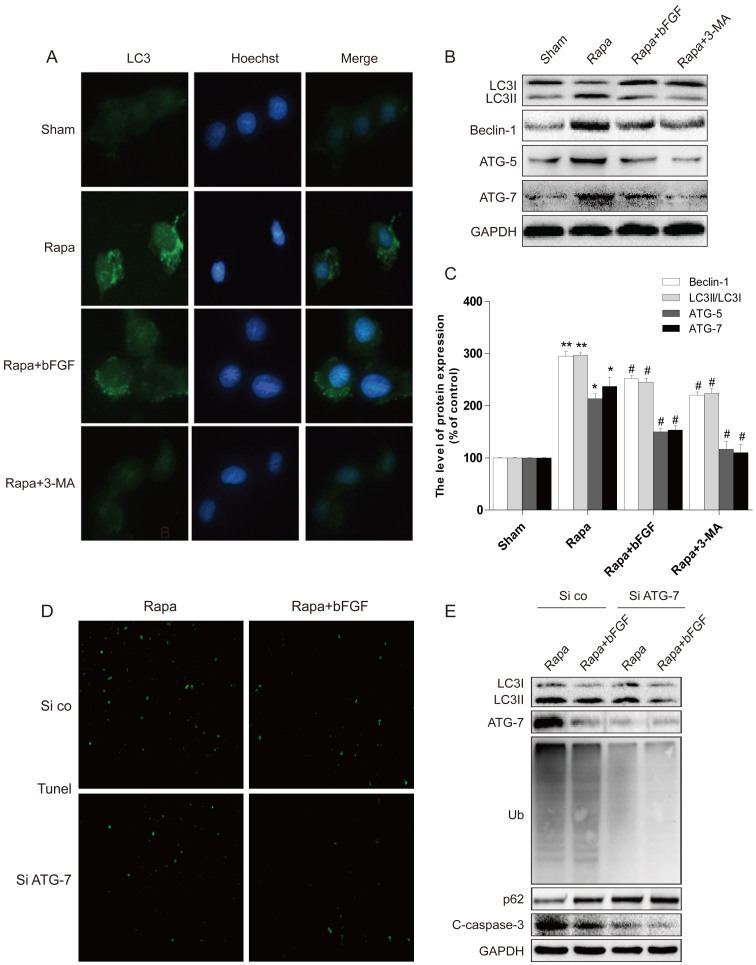
The cardioprotective effects of bFGF are related to the inhibition of autophagy induced by rapamycin. (A) Immunofluorescence staining results of LC3 (green), in which the nuclei are labeled with Hoechst (blue). (B, C) The protein expression of LC3II/LC3I, ATG-7, ATG-5 and beclin-1 in the sham, rapa group, and rapa treated with bFGF or 3-MA. The optical density analysis of LC3II/LC3I, ATG-7, ATG-5 and beclin-1 in the sham, rapa group, and rapa treated with bFGF or 3-MA. (D) H9C2 cells (si co: random siRNA, si ATG-7: ATG-7 siRNA) were treated with 100 ng/ml rapamycin with or without 40 ng/ml bFGF. The cells were collected and stained with TUNEL. (E) Protein expression of LC3II/LC3I ATG-7, Ub, SQSTM1/p-62 (Thr269/Ser272) and cleaved-caspase-3 of the rapamycin group, and rapamycin treated with bFGF in H9C2 cells (si co: random siRNA, si ATG-7: ATG-7 siRNA). * *P* < 0.05 versus the sham group, # *P* < 0.01 versus the rapamycin group. Rapa, rapamycin.

To further confirm our hypothesis that the effect of bFGF is related to the promotion of autophagy clearance in a cellular model, H9C2 cells were treated with rapamycin or combined with bFGF. Based on the fluorescence activated cell sorting analysis, rapamycin-induced cell apoptosis was restored after bFGF addition. Furthermore, the same response was detected in the group treated with the autophagy specific inhibitor, 3-MA, suggesting that bFGF exerts its beneficial effect via attenuating the autophagy in H9C2 cells caused by rapamycin (Fig. 6A and 6B). Next, we focused on the mechanisms of bFGF on autophagy. Cell immunofluorescence staining showed that the increase of LC3 puncta in the rapamycin-treated H9C2 cells group was inhibited when combined with bFGF (Fig. 7A), which is consistent with the results of the autophagy inhibitor, 3-MA. This was further conformed by western blot. Rapamycin incubation resulted in significant activation of LC3II, ATG-7, ATG-5 and beclin-1, which was previously inhibited by bFGF and 3-MA treatment (Fig. 7B and 7C). Collectively, these data indicate that autophagy inhibition by bFGF is involved both *in vitro* and in an animal model.

To further confirm our hypothesis that inhibition of autophagy is important for the protective effect of bFGF in rapamycin-induced apoptosis, siRNA was used to knockdown ATG-7 in H9C2 cells. Infection with shAtg7 encoding lentivirus reduced the Atg7 protein levels as shown Western blot compared to the negative control virus (Fig. 7D and 7E). Expression of shAtg7 lentivirus also reduced the conversion of LC3-I to LC3-II, validating the knockdown of autophagy. The shAtg7 lentivirus also upregulated p62 protein expression and inhibited Ub expression (Fig. 7E), which suggested that the protective role of bFGF is associated with the inhibition of excessive autophagy and the regulation of protein degradation via a p62-dependent pathway. Importantly, H9C2 cells with reduced autophagy showed improved survived compared to the controls for the bFGF and rapamycin co-treatment (Fig. 7D). Therefore, the genetic reduction of autophagy blocked the harmful effects of rapamycin, consistent with the hypothesis that cells treated with bFGF in the context of mTOR inhibition are dying due to excessive autophagy.

**Figure 8:**
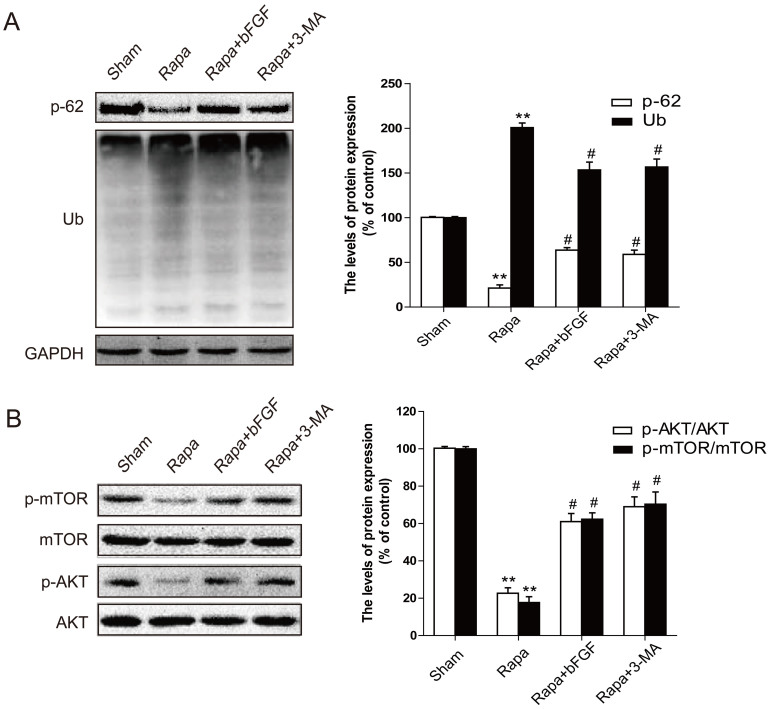
bFGF activates the Akt/mTOR signaling pathways and inhibits rapamycin-induced protein ubiquitination after 12 h treatment in H9C2 cells. (A) Protein expression and optical density analysis of Ub and SQSTM1/p-62 (Thr269/Ser272) of the sham, rapamycin group, and rapamycin treated with bFGF or 3-MA group in H9C2 cells. (B) Protein expression and the optical density analysis of p-AKT (Ser473) and p-mTOR (Ser2448) of the sham, rapamycin group, and rapamycin treated with bFGF or 3-MA group of H9C2 cells. * *P* < 0.05, ** *P* < 0.01 versus the sham group, # *P* < 0.05 versus the rapamycin group. Rapa, rapamycin.

Based on the above evidence, we then determined whether exogenous bFGF contributes to cell survival through clearance of poly-ubiquitinated protein accumulation *in vitro*. As indicated in Fig. 8A, poly-ubiquitinated protein expression increased with rapamycin and was suppressed by bFGF. Down-regulation of p62 was also alleviated by bFGF treatment. The p-Akt/Akt and p-mTOR/mTOR levels were then confirmed in H2C9 cells, and consistent with our *in vivo* study, we observed that both p-Akt and p-mTOR levels in the bFGF-incubated group markedly increased compared to the rapamycin group in the cell model (Fig. 8B). Taken together, these findings indicate that the protective role of bFGF in cardiac cell death is partially modulated through the PI3K/Akt/mTOR signaling pathways.

### p62 is required for Ubiquitinated protein clearance of bFGF

**Figure 9:**
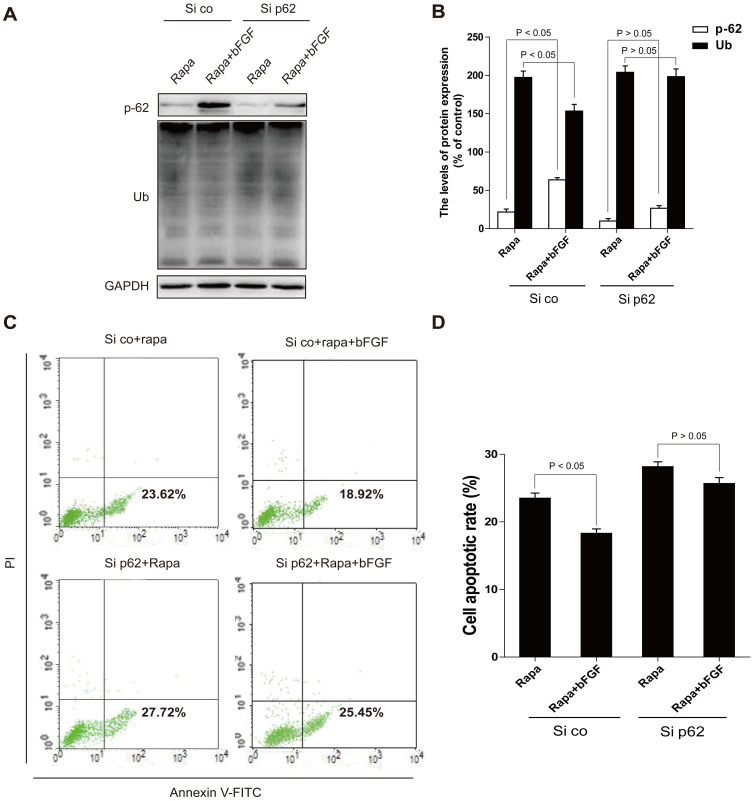
Silencing of p62 partially attenuates the anti-apoptosis effect of bFGF, whereas shRNA against the autophagic machinery Atg7 prolongs the survival of cells co-treated with bFGF and rapamycin. (A, B) Protein expression and optical density analysis of Ub and p62 of the rapamycin group, and rapamycin treated with bFGF in H9C2 cells (si co: random siRNA, si p62: p62 siRNA). (C, D) H9C2 cells (si co: random siRNA, si p62: p62 siRNA) were treated with 100 ng/ml rapamycin, with or without 40 ng/ml bFGF. The cells were collected and stained with annexin V-FITC/propidium iodide and detected by flow cytometry. The lower right panel indicates the apoptotic cells.

To elucidate the role of p62 in ubiquitinated protein clearance, H9C2 cells were treated with rapamycin combined with bFGF, and the levels of p62 were determined using immunoblotting. Consistent with the *in vivo* data, p62 protein expression was decreased and the Ub levels were increased with rapamycin treatment, and bFGF reversed the p62 and Ub levels. Because p62 may be required for ubiquitinated protein clearance of bFGF, we then used siRNA to knockdown p62, an essential component of the ubiquitination pathway, to further validate this result. As shown in Fig. 9A, the inhibition by p62-siRNA on p62 expression in H2C9 cells was confirmed. As expected, silencing of p62 markedly reduced the stimulation of intracellular p62 levels induced by bFGF, and fully abolished the ubiquitinated protein clearance effect of bFGF (Fig. 9A and 9B). In addition, bFGF decreased the rapamycin-induced cell apoptosis rate (18.30 ± 0.67% versus 22.83 ± 1.16%, *P* < 0.05), and after silencing p62, the cell apoptosis rates were almost unaltered by bFGF treatment (25.67 ± 0.88 versus 28.17 ± 0.76%, *P* > 0.05), indicating that bFGF protected against myocardial apoptosis in a p62-dependent manner (Fig. 9C and 9D). Collectively, these data further confirmed that the protective role of bFGF is associated with inhibition of excessive autophagy and the regulation of protein degradation via a p62-dependent pathway.

## Discussion

Many patients suffer from ischemic heart disease secondary to acute myocardial infarction. These are among the most prevalent health problems in the worldwide and are a major cause of morbidity and mortality. After ischemia, a long period of secondary myocardial injury occurs following myocardial reperfusion, including oxidative stress, inflammation, necrosis and apoptosis. The loss of myocardial cells is the main factor that interferes with recovery from the secondary damage. Several studies have shown that bFGF delivered during reperfusion protects from I/R injury^22^,^23^,^24^. However, as a multifunctional factor, the protective effect of bFGF on the autophagy-induced cell death observed in myocardial ischemia/reperfusion is not yet fully understood. In our previous study, we showed that administration of bFGF immediately after reperfusion significantly protected the heart from I/R-induced injuries^24^. In the present study, we treated myocardial I/R mice model with recombinant bFGF, and demonstrated the protective effect and molecular mechanism of bFGF in the autophagy-mediated ubiqutintation *in vivo* and *in vitro*.

Autophagy is an evolutionarily conserved process involved in the degradation of long-lived or damaged proteins and organelles^25^. Autophagy has been implicated in myocardial I/R, although the exact functional role of autophagy in the cell survival and death pathways associated with heart damage remains unclear. Several lines of evidence suggest that autophagy may promote cell survival by purging the cell of damaged proteins to generate the intracellular building blocks required to maintain vital functions during nutrient-limiting condition^26^,^27^. Autophagy may also promote cell death through excessive self-digestion and degradation of essential cellular constituents or it may interact with the apoptotic cascade in the heart^28^, indicating a double-edged sword for the biological function of autophagy in myocardial I/R injury. Many damaged cardiomyocytes showed features of autophagic/lysosomal cell death during myocardial I/R injury, including cytoplasmic autophagic vacuoles and the induction of GFP-LC3 immunofluorescence^29^. The present study showed increased TUNEL-positive myocardial cells (Fig. 1B), up-regulation of cleaved-caspase-3 protein (Fig. 1C), and enhancement of LC3 II and beclin-1 levels (Fig. 2) in the myocardial I/R animal model, which confirmed that autophagy-induced apoptosis was a primary event in the secondary damage of in myocardial I/R^26^,^30^. After bFGF treatment, the expression of the LC3II protein decreased significantly at 3 day after myocardial I/R (Fig. 2B). The immunofluorescence staining results showed that the LC3 puncta were increased in the I/R group compared to the sham injury group and that puncta were recovered by bFGF treatment (Fig. 2A). Notably, bFGF also inhibited autophagy induced by the autophagy sensitizer rapamycin and increased the viability in a H9C2 cell model (Fig. 6). The effect of bFGF was consistent with that of the classical autophagy inhibitor, 3-MA, and genetic knockdown of the autophagy gene Atg7 decreased cell death which induced by rapamycin treatment (Fig. 9E), suggesting that bFGF suppressed rapamycin-induced autophagy in cardiomyocytes. To the best of our knowledge, this is the first study demonstrating that bFGF protectes myocardial cells and improves recovery from I/R via the inhibition of excessive autophagy.

As a main downstream signal activated by bFGF, PI3K/Akt has an essential cardioprotective effect. The PI3K/Akt pathway is particularly important for mediating myocardial cell survival under a wide variety of circumstances. Activation of the PI3K/Akt pathways is essential for growth factor-mediated cell survival. Moreover, the downstream signals of PI3K/Akt/mTOR play an important role in programmed cell death, and phosphorylation of mTOR impairs myocardial cell death in myocardial I/R. In particular, previous reports suggest that phosphorylated mTOR provides cardioprotection by reducing autophagy and enhancing recovery in myocardial I/R^31^. As a macrolide anti-biotic, rapamycin is often used to induce autophagy via mTOR inhibition^32^. The mTOR-signaling pathway is considered a master regulator of multiple interrelated functions and mechanisms relevant to cell growth, proliferation and death. Cardiac mTOR overexpression in the heart is sufficient to provide substantial cardioprotection against I/R injury and suppress the inflammatory response^33^. In H9C2 cells, doxorubicin induction of ROS signals the Akt/mTOR pathway by activating positive regulators of PI3K; this leads to H9C2 cells apoptosis^34^. Furthermore, mTOR may directly modulate separate mechanisms controlling myocardial cell death, such as the pro-apoptotic molecules BAD and Bcl-2^35^. This dual role of mTOR may be based on the level of autophagy activation and/or various activated phases or situations. The mTOR pathway may have opposing effects on myocardial cell death mechanisms, which depend on the situation and stress level. These factors must be considered when designing potential therapies. In this study, we observed that autophagy was excessively activated during the early stage of I/R, and bFGF treatment stimulated phosphorylation of PI3K/Akt and further enhanced mTOR signaling (Fig. 4 and 8B), resulting in autophagy inhibition. In our H9C2 cell model, rapamycin combined with bFGF or the autophagy inhibitor, 3-MA, also showed increased cell viability compared to the rapamycin group. Additionally, genetic knockdown of the autophagy gene Atg7 decreased H9C2 cell death induced by rapamycin. Taken together, these findings suggest that the role of bFGF in myocardial cell death and recovery in I/R is involved in autophagy inhibition via activation of the Akt/mTOR signaling pathway.

In addition to its central roles in protein quality control, regulation of cell cycle, intracellular signaling, DNA damage response and transcriptional regulation, the ubiquitin-proteasome system (UPS) plays specific roles in the heart disease. Dysfunction of the UPS is associated with several myocardial diseases^36^, UPS-mediated protein degradation is an ATP-dependent sophisticate process that requires the collaboration of a large number of proteins involved in ubiquitination of the substrate protein, delivery of the ubiquitinated proteins to the proteasome, and proteasomal degradation. The proteasomal and lysosomal degradation were historically regarded as two parallel pathways, but emerging evidences suggest that the two pathways actually interact with each other and their interplay may be critical to maintaining proteostasis in the cell^37^,^38^. Rapamycin can regulates in vitro performance of the 20S proteasome, which is an essential intracellular protease of the ubiquitin-proteasome pathway. Rapamycin compromises degradation of the model protein, attenuates two of three major peptidase activities, and interferes with interactions of the 20S proteasome with its physiologic regulators^39^. Proteasome inhibition has been reported to activate macroautophagy in cultured cells and intact mice^40^. However, very few studies have examined the in vivo effect of inhibition of the bFGF on UPS function. UPS dysfunction leads to misfolded proteins aggregates, named ubiquitin^41^. Furthermore, excessive ubiquitinated protein accumulation in the etiology of the disease itself leads to UPS dysfunction. The p62 protein conveys ubiquitinated proteins to both proteasome and autophagy degradation systems. In particular, p62 was the first protein shown to bind directly to LC3 to facilitate autophagosome degradation of ubiquitinated protein aggregates^42^. As an ubiquitin-binding protein, p62 plays an important homeostatic role in clearing cells of misfolded proteins, performing a critical function in the formation of signaling complexes that activate NF-kB, p38 MAPK and PI3K. In our study, we found that bFGF enhanced the clearance of ubiquitinated proteins and significantly increased the expression of p62 in myocardial I/R heart. To further validate the role of p62 in the cardioprotective effect of bFGF, siRNA-p62 was used in H9C2 cells. As demonstrated by Figure 9, silencing of p62 partially inhibited the ability to clear ubiquitinated proteins of bFGF and reversed the anti-apoptosis effect of bFGF, indicating that the protective function of bFGF may be involved in the inhibition of excessive autophagic cell death and reversed toxic accumulation of ubiquitinated proteins via the p62 pathway. It should be noted that siRNA-p62 partly reversed the bFGF effect, indicating that autophagy clearance only represented part of the mechanism responsible for the myocardial preservation of bFGF.

In conclusion, bFGF significantly reduced the extent of damage after myocardial I/R injury, and improved the survival of cardiomyocytes. We first reported that the neuroprotective role of bFGF is related to the inhibition of excessive autophagy and enhanced ubiquitinated protein clearance via p62. Furthermore, activation of the downstream signaling pathways PI3K/Akt/mTOR is essential for the effect of bFGF in myocardial cell death both *in vivo* and *in vitro*. Our study demonstrates that therapeutic strategies using bFGF may be suitable for recovery from myocardial I/R injury.

## Methods

### Animal

Adult male C57/B6 mice (8 to 12 weeks of age) were supplied by the Animal Center of the Chinese Academy of Sciences. All protocols involving animals were approved by Wenzhou Medical University and the Institutional Animal Care and Use Committee; experiments involving animals were performed in accordance with the relevant approved guidelines and regulations.

### Reagents and antibodies

Dulbecco's modified Eagle's medium (DMEM) and fetal bovine serum (FBS) were purchased from Invitrogen (Invitrogen, Carlsbad, CA). Recombinant human bFGF was purchased from Sigma. Anti-Akt, p-Akt (Ser473), anti-mTOR, p-mTOR, cleaved-caspase-3, anti-LC3, ATG-7, ATG-5, anti-beclin-1 and GAPDH antibodies were purchased from Santa Cruz Biotechnology (Santa Cruz, CA, USA). Goat anti-rabbit and anti-mouse IgG-HRP was purchased from Cell Signaling Technology, Inc. (Danvers, MA, USA). An enhanced chemiluminescence (ECL) kit was purchased from Bio-Rad (Hercules, CA, USA). Rapamycin, the autophagy inhibitor 3-Methyladenine and all other reagents were purchased from Sigma unless otherwise specified.

### Cell Culture and Viability Assay

Rat cardiomyocyte H9C2 cells were purchased from the American Type Culture Collection. The Cells were cultured in DMEM (Invitrogen) and supplemented with heat-inactivated 10% FBS (Invitrogen), 5% horse serum, and antibiotics (100 units/ml penicillin, 100 μg/ml streptomycin). They were then incubated in a humidified atmosphere containing 5% CO_2_ at 37°C. Based on our previous study, the cells were treated with rapamycin (100 nM), bFGF (40 ng/ml), bFGF with rapamycin or the autophagy inhibitor 3-methyladenine (3-MA, 5 mM; Sigma-Aldrich) with rapamycin. All experiments were performed in triplicate.

### Myocardial I/R model in mice and bFGF treatment

Experimental myocardial I/R was induced by transient myocardial ischemia for 30 min and was followed by reperfusion for 3 days, as described previously. The mice were anesthetized with 4% chloral hydrate (100 mg/kg, ip) and placed on a ventilator (Harvard Rodent Ventilator, Harvard Apparatus, Holliston, MA) in the right lateral decubitus position, and the core temperature was maintained at 37°C with a heating pad. After a left lateral thoracotomy and pericardiectomy, the left anterior descending coronary artery was occluded for 30 min with an 8-0 nylon suture and polyethylene tubing to prevent arterial injury and then reperfused for 3 days. The mice that survived surgery were assigned randomly to the different treatment groups (n = 6 to 15 per group). Control group operations were performed in which the animals underwent the same surgical procedure without coronary artery ligation (n = 6 to 8 per group). The I/R mice were administered 2 μg bFGF/mouse (intravenous injection) or 10 mg/kg rapamycin (intraperitoneal injection) at 30 min after ischemia.

### Echocardiography

The mice underwent transthoracic echocardiography using an ultrasound system with a 12 MHz phased-array SONOS-7500 transducer (PHILIPS, the Netherland). The mice were imaged in the left lateral decubitus position 3 days post-infarction. M-mode echocardiograms were obtained from the parasternal long axis views and short axis views at the papillary muscle level. The left ventricular dimension at end-diastole and end-systole (LVEDd, LVEDs) were measured. The ejection fraction (EF) and fractional shortening (FS) were then calculated as follows: EF = 100 × stroke volume/end-diastolic volume. FS = 100 × (LVEDd − LVEDs)/LVEDd. All data were measured in at least three consecutive cardiac cycles and were performed by an experienced technician who was blinded to the group.

### TUNEL Assay

DNA fragmentation in vivo was detected using a one-step TUNEL Apoptosis Assay KIT (Roche, Mannheim, Germany). The images were captured with a Nikon ECLIPSE Ti microscope (Nikon, Japan). The apoptotic rates were measured using a PI/Annexin V-FITC kit (Invitrogen, Carlsbad, CA, USA) and then analyzed by a FACScan flow cytometer (Becton Dickinson, Franklin Lakes, NJ, USA) according to the kit's manual.

### Fluorescence activated cell-sorting (FACS) analysis

The cells were cultured at a density of 2 × 10^5^ cells per well in growth medium for 24 h in 6-well plates. The cells were treated with rapamycin (100 nM), bFGF (40 ng/ml), or bFGF with rapamycin or the autophagy inhibitor 3-methyladenine (3-MA, 5 mM; Sigma-Aldrich), with rapamycin. Annexin V assays were performed using the Annexin V-FITC Apoptosis Detection Kit (Becton Dickinson, San Jose, CA). The cells were washed twice with cold phosphate-buffered saline (PBS) and resuspended in binding buffer before the addition of Annexin V-FITC and propidium iodide (PI. Cells were vortexed and incubated for 15 min in the dark at room temperature before analysis using a FACSCalibur flow cytometer (BD Biosciences, San Jose, CA) and FlowJo software (Tree Star, San Carlos, CA).

### Masson staining

Mice were sacrificed at 3 days post-infarction and hearts were harvested following the established methods. Harvested hearts were either fixed in 10% formalin and embedded in paraffin for hematoxylin and masson staining or frozen in OCT compound for all other assessments. Specimens were serially sectioned at 8 mm thickness from apex to the ligation level (approximately 0.5 mm in length). For observation of fibrosis, Masson's trichrome kit (IMEB, San Marcos, CA) was used to stain collagen fibers. The area of collagen deposition was measured by ImageJ and the extent of fibrosis was determined by dividing the area of collagen deposition by the entire cardiac tissue area. Twelve randomly selected sections from each group were utilized for quantification.

### Immunofluorescence Staining

To determine the LC3, p-62 and Ub activities, sections were incubated with 0.3% H_2_O_2_ in methanol for 30 min, followed by blocking with 1% bovine albumin in PBS for 1 h at room temperature. Next, the sections were incubated at 4°C overnight with a primary antibody against LC3 (1:200), p-62 (1:200) or cleaved Ub (1:200). After primary antibody incubation, the sections were washed for 4 × 10 min at room temperature and then incubated with donkey anti-mouse/rabbit, donkey anti-rabbit/mouse, or donkey anti-goat secondary antibody (1:500; Invitrogen) for 1 h at room temperature. The saline injection group was the negative control. The images were captured using a Nikon ECLPSE 80i.

### Western blot analysis

Total proteins were purified using protein extraction reagents for the heart tissue and H9C2 cells. The equivalent of 50 μg of protein was separated on a 12% gel and then transferred onto a PVDF membrane. After blocking with 5% fat-free milk, the membranes were incubated with the relevant protein antibodies overnight. The membranes were washed with TBS and treated with secondary antibodies for 2 h at room temperature. The signals were visualized with the ChemiDicTM XRS + Imaging System (Bio-Rad Laboratories, Hercules, CA, USA), and the band densities were quantified with Multi Gauge Software of Science Lab 2006 (FUJIFILM Corporation, Tokyo, Japan).

### Transfection of P62 or ATG-7 small interfering RNA (siRNA)

The cells (5 × 10^5^ cells/well) were seeded in 6-well plates and transfected with siRNA using Opti-MEM media (Gibco by Life Technologies). Briefly, 4.0 μg of P62 or ATG-7 siRNA was mixed with Opti-MEM media. Separately, Lipofectamine™ 2000 reagent (Invitrogen) was mixed with Opti-MEM and then the mixtures were combined for 25 min at room temperature. The mixture was then added to each well containing cells and medium and incubated for 4–6 hours, then changed into antibiotic-free DMEM for 24 h. The cell media was changed to antibiotic-free media prior to the addition of siRNA.

### Statistical Analysis

Data are expressed as the mean ± SEM. Statistical significance was determined using Student's t-test when there were two experimental groups. When more than two groups were compared, statistical evaluation of the data was performed using one-way analysis of variance (ANOVA) and Dunnett's post-hoc test. *P* values < 0.05 were considered statistically significant.

## Author Contributions

J.X., Z.-G.W. and X.-K.L. conceived and designed the experiments. Y.W., Z.-G.W., K.-T.J., Q.L., L.Z., Y.H., Y.-L.L., H.-Y.Z., D.H. and W.-K.F. performed the experiments. M.-P.C., X.-B.F., Z.-G.W. and J.X. analyzed the data. J.X. and M.-P.C. wrote the paper.

## Figures and Tables

**Table 1:** Echocardiographic assessment suggests that bFGF improves cardiac function

Parameters	Sham	I/R	I/R+bFGF
LVEDd	3.0 ± 0.1	3.4 ± 0.2*	3.1 ± 0.1#
LVESd	1.3 ± 0.1	2.1 ± 0.3*	1.6 ± 0.1#
EF(%)	92.0 ± 2.3	72.9 ± 1.4**	83.1 ± 2.5#
FS(%)	58.4 ± 2.8	37.6 ± 1.6**	45.7 ± 1.8#

LVEDd: left ventricular end diastolic dimension. LVESd: left ventricular end systolic dimension. EF: left ventricular ejection fraction. FS: left ventricular fractional shortening. Values are mean ± SD,

*represents *P* < 0.05 vs. Sham group.

**represents *P* < 0.01 vs. Sham group.

^#^represents *P* < 0.05 vs. I/R group.
